# Prediction of assisted reproductive technique outcome in elevated early follicular phase follicle stimulating hormone with Mullerian inhibiting substance level

**Published:** 2012-05

**Authors:** Leili Safdarian, Zahra Khayatzadeh, Ebrahim Djavadi, Atossa Mahdavi, Marzieh Aghahosseini, Ashraf Aleyasin, Parvin Fallahi, Sima Khayatzadeh, Arash Ahmadzadeh, Mohhamad Bagher Larijani

**Affiliations:** *Department of Obstetrics, Gynecology and Infertility, Shariati Hospital, Tehran University of Medical Sciences, Tehran, Iran.*

**Keywords:** *Anti-mullerian hormone*, *In-vitro fertilization*, *Assisted reproductive technique*, *Inhibin B*

## Abstract

**Background:** Detection of best predictor of ovarian reserve in patients with temporarily or consistently elevated early follicular phase serum levels of FSH is one of the most important goals in assisted reproductive technique (ART).

**Objective:** To evaluate whether high level of anti-mullerian hormone level is related to success of ART in patients with temporarily or consistently elevated early follicular phase serum levels of FSH.

**Materials and Methods:** Sixty three women underwent intracytoplasmic sperm injection (ICSI) with GnRH-agonist long protocol or intrauterine insemination (IUI) in a prospective cohort study. FSH, inhibin B and anti-Mullerian hormone (AMH) levels were measured in these women whom were divided to three groups (persistently elevated FSH, variably elevated FSH and, normal FSH level). Basal characteristics, stimulation parameters, and pregnancy occurrence were evaluated.

**Results:** AMH was significantly higher in women with persistently elevated early follicular phase FSH achieving pregnancy. Women with normal FSH did not have significant difference in AMH level between conceived and non conceived cycles. Women with only one elevated early follicular phase FSH achieving pregnancy did not have significant difference in AMH level with non pregnant women. Response to gonadotropin stimulation, recommendation to oocyte donation significantly differed between the groups.

**Conclusion:** This study has demonstrated that relatively young women with persistently or intermittently elevated day 3 FSH levels have diminished ovarian reserve and lower ART success. However, in women whose FSH levels were constantly elevated, AMH (not inhibin B) concentrations were significantly higher in ART cycles resulting in pregnancy. Therefore, AMH level is a good predictor of ART outcome in patients with elevated early follicular phase serum levels of FSH.

## Introduction

Assessment of “ovarian reserve” is of high value before infertility treatment is undertaken. Identification of both low and high responders prior to ovarian stimulation allows physicians to optimize stimulation protocols in order to decrease cycle cancellation rate and side effects, such as ovarian hyperstimulation syndrome. Despite available ovarian reserve predictors [such as early follicular phase follicle-stimulating hormone (FSH), estradiol (E_2_), inhibin B levels, ovarian volume and antral follicle count (AFC)] (1) anti-Mullerian hormone (AMH) provides a good prognostic value for clinical purposes (2). Anti-Mullerian hormone (AMH) is a dimeric glycoprotein, a member of the transforming growth factor-beta superfamily produced by granulosa cells, from pre-antral and antral follicles (3) and according to Visser and Themmen “the main physiological role of AMH in the ovary seems to be limited to the inhibition of the early stages of follicular development” (4). 

AMH is produced independently from FSH and it causes inhibition of FSH-induced follicular growth. It is also stated that “it has a direct autocrine-paracrine effect on the granulosa cells, oocyte function and embryo quality” (5, 6). AMH production is independent of FSH and inhibits FSH-induced follicular growth. It also has a direct autocrine-paracrine effect on the granulosa cells, oocyte function and embryo quality (5, 6). As a marker for ovarian reserve, MIS/AMH correlates positively with ovarian response to COH in normoovulatory women, but in poly cystic ovary patients (PCOS) AMH may not be an accurate predictor for pregnancy (7).

Within the ovary, inhibin B is produced by granulosa cells of pre-antral and early antral follicles, structurally similar to AMH, in the transforming growth factor-beta superfamily that selectively inhibits pituitary FSH release (8). Lower serum inhibin B is associated with poor response to gonadotropins, higher IVF cycle cancellation lower number of retrieved oocytes, and significantly reduced pregnancy rate (9), although; inhibin B was noted to have a high false-positive rate, causing inappropriate patient exclusion from IVF treatment (10). 

Elevated early follicular phase FSH is frequently observed in subfertile patients. In these women, temporary normalization of FSH concentrations is known to occur, although ovarian reserve may be different in comparison to age matched women but normal FSH levels. According to Koning et al. the endocrine cycle profile in younger subfertile patients with consistently elevated basal FSH resembles that in published data from older women and also reflects a low ovarian reserve. They conclude that “Normalization of FSH in association with normal inhibin B suggests a temporary increase of the available cohort” (11).

Elevated early follicular phase serum levels of FSH have been supposed to be a dilemma in counseling our infertile patients in our department, because it is frequently associated with poor ovarian response during gonadotropin stimulation cycles.

However; there exists an important unanswered question, which will benefit from our treatment protocols? The exclusion of extremely reduced ovarian reserve couples from ART could effectively reduce costs for the health system. Moreover useless medical treatments, surgical risks, stress and disappointment could be avoided. Due to few available studies in this regard (11) we conducted this novel study in order to detect the best predictor of ovarian reserve in these patients with temporarily or consistently elevated early follicular phase serum levels of FSH.

## Materials and methods

This prospective cohort study was conducted on consecutive patients, referred to Shariati Hospital Infertility Clinic, affiliated to Tehran University of Medical Sciences. The study was initiated after achieving written consents of the participants, during the year 2008. The patients were screened on day 3 of the menstrual cycle. The patients with basal FSH values ≥10 IU/l show very poor outcome with respect to the number of oocytes retrieved and pregnancy rate. All patients with regular menstrual cycles (21-35 days) but day 3 FSH values of ≥10 IU/l were participated in the study (50 women entered the project). 

All women had normal endocrine screen, no current medical illness, no history of previous abortions, and no history of ovarian surgery, any current use of oral contraceptives and any pregnancy or breastfeeding in the past 6 months. Exclusion criteria was patients with age ≥41 years, poly cystic ovarian syndrome (PCOS), egg or embryo donations, endometriosis, uterine myoma, tubal infertility due to hydrosalpinx, testicular sperm extraction (TESE) or percutaneous epididymal sperm aspiration (PESA). 

AMH, inhibin B and second FSH concentrations on day 3 of the study cycle were measured. In this second laboratory measurement, 19 patients had FSH values ≤10 IU/l and 31 patients had FSH values ≥ 10 IU/l, respectively. For better comparison; a third group of controls (13 women with similar characteristics) and cycle day 3 values of less than 10 IU/l were added to our study. Fifty patients with regular menstrual cycles but day 3 FSH values of ≥10 IU/l and 13 women with similar characteristics and cycle day 3 values of less than 10 IU/l (as control group) were participated in the study. For each participant a questionnaire was filled by the researchers. Data were collected from questionnaires, clinical, laboratory notes and ultrasound reports.

All the participants received folic acid 1mg/day (Ruz Daru Co, Iran) before initiating the induction cycle. Thirty seven patients entered intracytoplasmic sperm injection and embryo transfer (ICSI-ET) and 26 patients entered intrauterine insemination (IUI) due to inadequate follicular response and serum estradiol level of less than 400 pg/ml or patient inquiry due to financial problems.

In the ICSI-ET group, patients received low dose oral contraceptive pills (Iran Hormone Co, Iran) on day 3 of the previous cycle and doxycycline (Razak Co, Iran) 100 mg twice a day for the first 10 days of the previous cycle. Then a long term desensitization protocol using the GnRH agonist Buserelin 500 micrograms subcutaneously was started on the day 21 of the previous cycle. 

After complete desensitization, ovarian stimulation using HMG (Ferring, Germany) was commenced on day 3 of the next cycle according to patient’s age, ovarian reserve, body mass index, previous response to gonadotropins at a daily dose of 300-450 IU. Transvaginal ultrasound (Siemens, Sonoline G20) using a 7.5 MHz transvaginal probe by the same attending physicians was done every 3 days for measuring follicular development and serum estradiol level was measured every 2-3 days by radioimmunoassay method; so gonadotropin dosage was adjusted. 

Final oocyte maturation triggered when at least 2 follicles with diameter of at least 17 mm was observed, with HCG (Ferring, Germany) 10000 IU administered as a single intramuscular injection. Oocytes were collected 36-38 hours later using transvaginal guided follicle aspiration under general anesthesia. After fertilization through intracytoplasmic sperm injection (ICSI), 3 high grade embryos were transferred transcervically 3 days later. Luteal phase support was started the day after ovum pick up by administration of progesterone suppository Cyclogest (Actavis, UK), 800 mg daily. Chemical pregnancy was detected by serum β-HCG analysis 14 days after embryo transfer and transvaginal ultrasound scan was scheduled 2 weeks later to confirm the diagnosis of clinical pregnancy. Non pregnant women discontinued their luteal phase support.

When adequate follicular response was not detected and serum estradiol level was reported to be less than 400 pg/ml IUI was performed and luteal phase support was started on that day by administration of progesterone suppository Cyclogest (Actavis, UK), 400 mg daily. In cases with adequate response, IUI was performed due to financial problems. Chemical pregnancy was detected by serum beta-HCG analysis 14 days after insemination and transvaginal ultrasound scan was scheduled 2 weeks later to confirm the diagnosis of clinical pregnancy. Non pregnant women discontinued their luteal phase support.

The study was performed in accordance with current guidelines on good practice in clinical research and the Declaration of Helsinki. The project was approved by the ethical committee of the infertility department of the university and was initiated after achieving written consents of the participants.

**Hormone assay**

Estradiol was measured by ELISA. DRG (Germany) lot N.42 KO28-4/2. LH was measured by ELISA. Pishtaz Teb Company Lot N. 87003. FSH was measured by ELISA. Pishtaz Teb Company Lot N. 87005. MIS/AMH (ng/ml) was measured by DSL (USA) lot. N. 891042. Active inhibin B (pg/ml) was measured by DSL (USA) lot N. 890885.

**Statistical analysis**

The parameters relevant to demographic and COH characteristics were presented based on median and range. The Statistical Package for Social Sciences (SPSS, version 12.0 for windows, SPPS Inc., Chicago, IL) was utilized for data collection and analysis. Normality was assessed using Kolmogorov-Smirnov test. T-test and Chi-square test were used for analysis as needed. p value less than 0.05 was considered for statistical significance.

## Results

63 patients enrolled in this prospective cohort study. Basal characteristics and hormone levels of the subjects are shown in table I. No differences were found between age, body mass index (BMI) and duration of infertility. Though FSH, LH, Estradiol, MIS, inhibin B levels differed significantly between the three groups. Table II shows the statistics of oocytes retrieved and embryos in patients responding to gonadotropin stimulation and subsequent ovum pick up operation. Retrieved oocytes, mature oocytes, pronucleous formation, fertilization rate, implantation rate had no significant differences.

**Figure 1:**
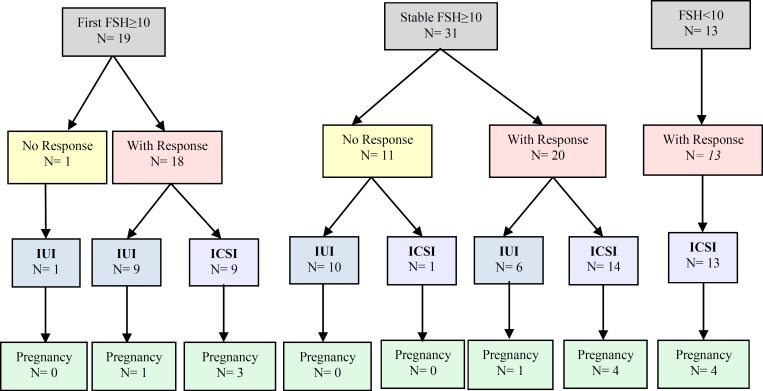
Algorithm for pregnancy rate according to treatment response in groups under study

In contrary to inhibin B, MIS was significantly higher in women with persistently elevated early follicular phase FSH achieving pregnancy (Table III). Women with Normal FSH did not have significant difference in MIS level between conceived and non-conceived cycles. Women with only one elevated early follicular phase FSH achieving pregnancy did not have significant difference in MIS level with non-pregnant women. Response to gonadotropin stimulation, oocyte donation consideration significantly differed between the comparison groups (Table IV). Figure 1 shows pregnancy rate according to treatment response in the groups under study. Nine patients with ovarian response in the first FSH ≥10 and six patients with ovarian response in the stable FSH ≥10 entered IUI cycle due to patient inquiry, mainly financial problems.

**Table I:** Baseline characteristics of the comparison groups

	**First FSH≥10** **N=19**	**Stable FSH≥10** **N=31**	**FSH<10** **N=13**	**p-value**
Age (years)	26 (20-38 )	28 (21-41)	28 (20-39)	0.727
Infertility duration (years)	5 (1-17)	3 (1-25)	5 (4-12)	0.285
BMI (kg/m^2^)	27 (22-34)	26 (17-33)	25 (23-30)	0.366
FSH (IU/L)	7.6 (4.1–9.7)	12 (10-61)	7.2 (4.1-8)	<0.001
LH (IU/L)	2.2 (0.4-9.7)	3.2 (1.1-71)	3(1-10)	0.002
E2 (pg/ml)	36 (11.3-88)	29.3 (7.7-642)	40(20-80)	0.112
MIS (ng/ml)	1.9 (0.4-14.8)	1.2 (0.01-10.9)	4.4 (0.2-7.4)	0.004
Inhibin B (pg/ml)	41.3 (0.1-117)	47 (0.1-156)	120.6 (38.9-150.5)	<0.001
Gonadotropin ampoules	25 (10-91)	38 (10-58)	25 (18-38)	0.011
Stimulation days	10 (8-13)	10 (7-13)	9 (8-10)	0.020

Values are presented as median and range. P-value less than 0.05 were considered for statistical significance.

BMI: body mass index.

FSH: follicle stimulating hormone (measured on the third day of the cycle).

LH: luteinizing hormone (measured on the third day of the cycle).

E2: Estradiol, (measured on the third day of the cycle).

MIS: mullerian inhibiting substance.

**Table II:** Stimulation characteristics of the comparison groups entering ICSI-ET

	**First FSH≥10** **N= 9**	**Stable FSH≥10** **N=15**	**FSH<10** **N=13**	**p-value**
Oocytes retrieved	9 (5-15)	12 (2-16)	8 (2-15)	0.604
MII oocytes	6 (3-11)	7 (1-15)	6 (2-14)	0.922
Injected oocytes	7 (3-11)	7 (1-15)	6 (2-15)	0.814
Pronucleous	6 (3-10)	4 (0-11)	5 (1-10)	0.472
Fertilization rate (%)	86.6%	73.3%	77.3%	0.120
Grade 1 embryos	4 (1-7)	3 (0-8)	1 (1-3)	0.001
Transferred embryos	4 (1-5)	4 (0-6)	2 (1-5)	0.253
Implantation rate (%)	21.2 %	15.1%	17.1%	0.767

Values are presented as median and range. P-value less than 0.05 was considered for statistical significance.

**Table III:** Comparison of MIS and inhibin B levels between pregnant and non-pregnant women

		**First FSH≥10** **N=19**	**Stable FSH≥10** **N=31**	**FSH<10** **N=13**	**Total** **N=63**
MIS (ng/ml)					
	Pregnant	4.5 (0.8-5.7)	4.3 (1.3-6.7)	4.1 (2.5-7.4)	4.3 (0.8-7.4)
	Non pregnant	1.8 (0.4-14.8)	0.6 (0.01-10.9)	4.4 (0.2-6.9)	1.5 (0.01-14.8)
	p-value	0.582	0.009	0.757	0.01
Inhibin B (pg/ml)					
	Pregnant	52.8 (0.1-66.6)	57.5 (40.4-156)	100.5 (38.9-120.6)	64.4 (0.1-156)
	Non pregnant	38.2 (0.1-117)	41.5 (0.1-120.4)	132.2 (80-150.5)	53.7 (0.1-150.5)
	p-value	0.764	0.126	0.089	0.262

Values are presented as median and range. P-value less than 0.05 was considered for statistical significance.

**Table IV:** Comparison of response rate between three groups

	**First FSH≥10** **N= 19**	**Stable FSH≥10** **N=31**	**FSH<10** **N=13**	**p-value**
Response rate	18 (94.7%)	20 (64.5%)	13 (100%)	0.004
Candidate for oocyte donation	1 (5.3%)	10 (32.3%)	0	0.009
Pregnancy rate	4 (21.1%)	5 (16.1%)	4 (30.8%)	0.553

## Discussion

This study has demonstrated that relatively young women with persistently or intermittently elevated day 3 FSH levels have diminished ovarian reserve and lower ART success. However, in women whose FSH levels were constantly elevated, MIS (not inhibin B) concentrations were significantly higher in ART cycles resulting in pregnancy. Therefore, inhibin B unlike AMH could not predict pregnancy in ART cycles of women with elevated FSH levels. Response rate and pregnancy rate was lower in persistently elevated FSH and candidates for oocyte donation were more in this group of patients.

Predicting ovarian response to gonadotropins and subsequent pregnancy is a logical question must be answered to all women before entering an IVF program. On the other hand exclusion of extremely reduced ovarian reserve women from ART could effectively reduce costs for our health system. AMH or MIS as a novel, stable and consistent predictor of ovarian reserve show a reduction throughout reproductive life and is undetectable after menopause (14-16). Similarly, early ovarian aging and premature ovarian failure have been associated with very low or undetectable serum levels, respectively (11, 17). Furthermore AMH levels do not significantly change during the menstrual cycle (18-20), whereas all other hormones secreted by the ovary show significant variations throughout the cycle. 

AMH seems to be a better marker in predicting ovarian response to controlled ovarian stimulation than age of the patient, FSH, estradiol and inhibin B. As Lamarca *et al* state “In clinical practice, AMH measurement may be useful in the prediction of poor response and cycle cancellation and also of hyper-response and ovarian hyperstimulation syndrome” (12). In our research we did not find any positive point about the Inhibin B, probably it is attributed to small sample size. 

According to a recent research, follicular fluid AMH level was significantly higher in good quality embryos and fertilized group (21). In young patients, the elevated FSH is most likely the result of diminished ovarian feedback from a smaller available cohort of follicles in relation to limited oocyte reserve. In these group of women whose early follicular FSH levels are elevated, counseling about the risk/benefit of the treatment, reducing the cost by denying treatment to bad prognosis couples and individualizing treatment strategy are warranted. Although basal AMH does not seem to predict pregnancy or non-pregnancy, but simply enables patients to be identified as being at a low or high probability of pregnancy after IVF (13).

In our young under study population (medians of 26 and 28 years) inhibin B and MIS level were higher in the group with persistently normal FSH level undergoing ART (Table I). Our research provides novel information about women whose FSH levels were constantly elevated; MIS (not inhibin B) concentrations were significantly higher in ART cycles resulting in pregnancy (Table II). Regarding stability and consistency of MIS levels, this study may consider MIS as the most reliable marker of ovarian aging and ovarian response to controlled ovarian hyperstimulation. Although pregnancy rate did not differ significantly (may be attributed to small sample size) between the research groups, but response rate and oocyte donation candidates differed significantly between two groups (Table III). Also, we postulated that temporally normalization of FSH is a reflection of advanced follicular development.

According to the results, women with first FSH ≥10 had more ovarian response and pregnancy than stable FSH ≥10 and women with persistently normal FSH had the highest response and pregnancy. This is in accordance with previous researches (11). In women whose FSH levels were constantly elevated, MIS concentration was significantly higher in ART cycles resulting in pregnancy. Also this was true for the group with stable FSH ≥10, but did not reach to a statistically significant result.

According to our knowledge, patients without ovarian response did not achieve pregnancy with IUI in both groups of first FSH ≥10 and stable FSH ≥10. As it is expected, in women with persistently normal FSH, IUI was not performed. Approximately half of the patients with ovarian response in the first FSH ≥10 became candidates for IUI and a third of patients with ovarian response in stable FSH ≥10 became candidates for IUI. Although, one pregnancy occurred in either groups, but it must be mentioned that the reason for IUI was mainly patients desires due their problems. However this point may cause some bias, but doesn’t decrease the value of the interesting research. In stable FSH≥10 group a patient without ovarian response requested ICSI and negative result was observed as it was predicted.

This study is a pilot research which has the novel idea to evaluate MIS level in women with temporary or persistently elevated FSH levels to predict the chance of pregnancy in these groups of diminished ovarian reserve. Considering our limitations such as small sample size, lager prospective observational studies are required to validate our results. Consequently individualization of treatment strategies in order to possibly reduce incidence of the clinical risks of ART, cycle cancellation and health costs along with optimized treatment burden is mandatory.
